# Giant retroperitoneal mucinous adenocarcinoma of the appendix: a case report

**DOI:** 10.1093/jscr/rjaf982

**Published:** 2025-12-13

**Authors:** Ari Solomon, Diego Oliver-Monasterio, Graal Diaz, Karim Jreije

**Affiliations:** Department of Graduate Medical Education, General Surgery, Community Memorial Hospital, 147 Brent St., Ventura, CA 93003, United States; Department of Graduate Medical Education, General Surgery, Community Memorial Hospital, 147 Brent St., Ventura, CA 93003, United States; Department of Surgery, General Surgery, Ventura County Medical Center, 300 Hillmont Ave, Ventura, CA 93003, United States; Department of Surgery, General Surgery, Ventura County Medical Center, 300 Hillmont Ave, Ventura, CA 93003, United States

**Keywords:** mucinous adenocarcinoma, retroperitoneal mass, pseudomonas peritonei, hyperthermic intraperitoneal chemotherapy (HIPEC)

## Abstract

Giant retroperitoneal mucinous adenocarcinoma of the appendix is a rare, aggressive malignancy characterized by mucin production and significant mass formation, often complicating early diagnosis due to nonspecific symptoms such as abdominal discomfort. Surgical resection, ranging from appendectomy to more extensive procedures like right hemicolectomy, is the primary treatment approach. This case presents a 53-year-old male with a 38 cm retroperitoneal mucinous adenocarcinoma of the appendix. The patient presented with right-sided flank pain, and subsequent imaging revealed a rapidly enlarging mass. Surgical resection was performed successfully via en-bloc resection with a right hemicolectomy. Postoperative care involved monitoring for complications, with the patient recovering uneventfully. This case highlights the challenges in diagnosing and managing large mucinous neoplasms and the importance of a multidisciplinary approach in ensuring optimal patient outcomes.

## Introduction

Giant retroperitoneal mucinous adenocarcinomas of the appendix are a rare and aggressive malignancy. Their rarity, comprising ~0.01%–0.2% of all gastrointestinal neoplasms, further contributes to diagnostic difficulty, as clinicians may not suspect appendiceal origins in patients presenting with abdominal masses or acute symptoms [1, 2]. This form of cancer arises from the appendix and is characterized by mucin production, leading to a large mass, sometimes filling the abdominal cavity or extending into retroperitoneal spaces. These tumors constitute ~55%–60% of all primary appendiceal carcinomas [3]. Mucinous adenocarcinomas of the appendix often present with nonspecific symptoms such as abdominal distention, pain, or digestive issues, making early diagnosis difficult [4].

Treatment typically involves surgical resection ranging from an appendectomy to a hemicolectomy, sometimes followed by chemotherapy [4, 5]. The goal of these interventions is to remove the tumor and reduce recurrence risks. Despite surgical advancements, the prognosis for patients with advanced disease remains poor, especially with metastasis or rupture. This case report describes a 53-year-old male found to have a 38 cm retroperitoneal mucinous adenocarcinoma of the appendix. The work has been reported in line with the SCARE criteria [6].

## Case report

**Figure 1:**
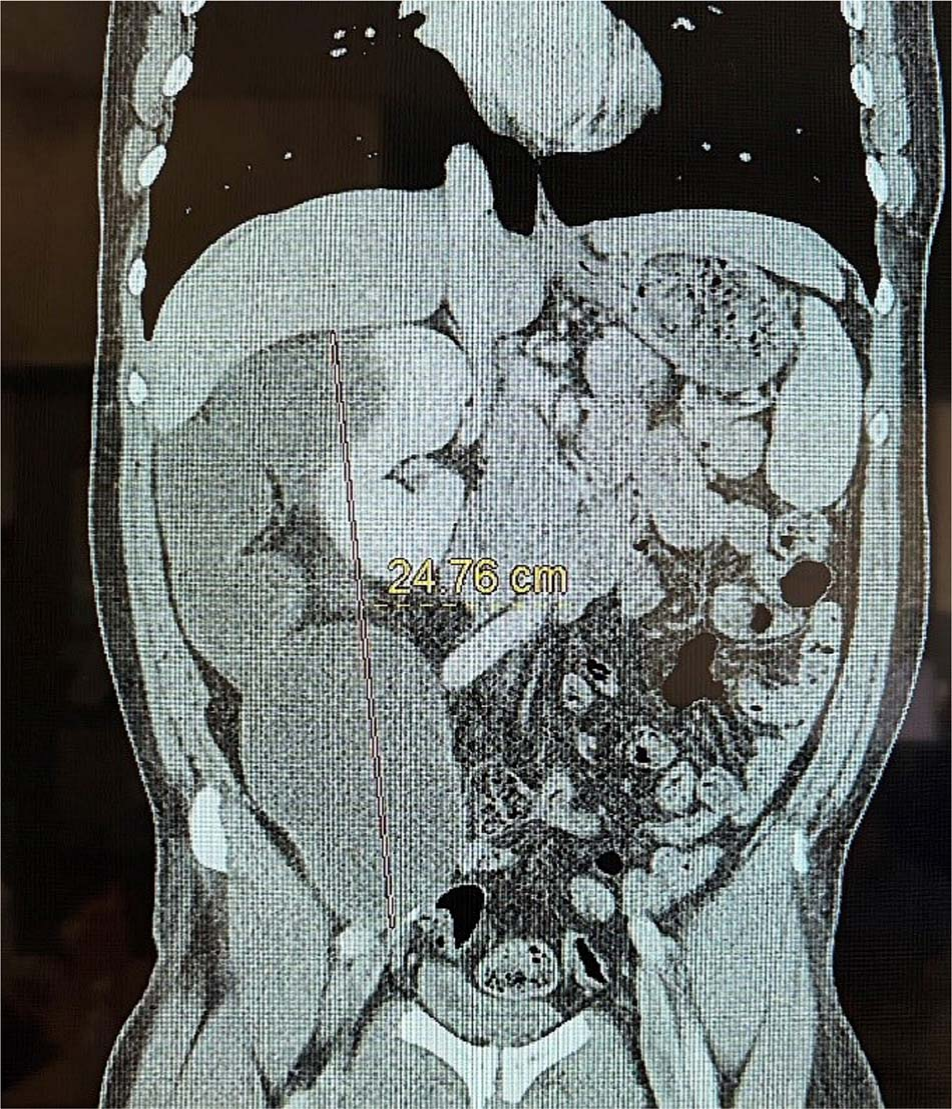
Coronal CT of the mucinous adenocarcinoma.

**Figure 2:**
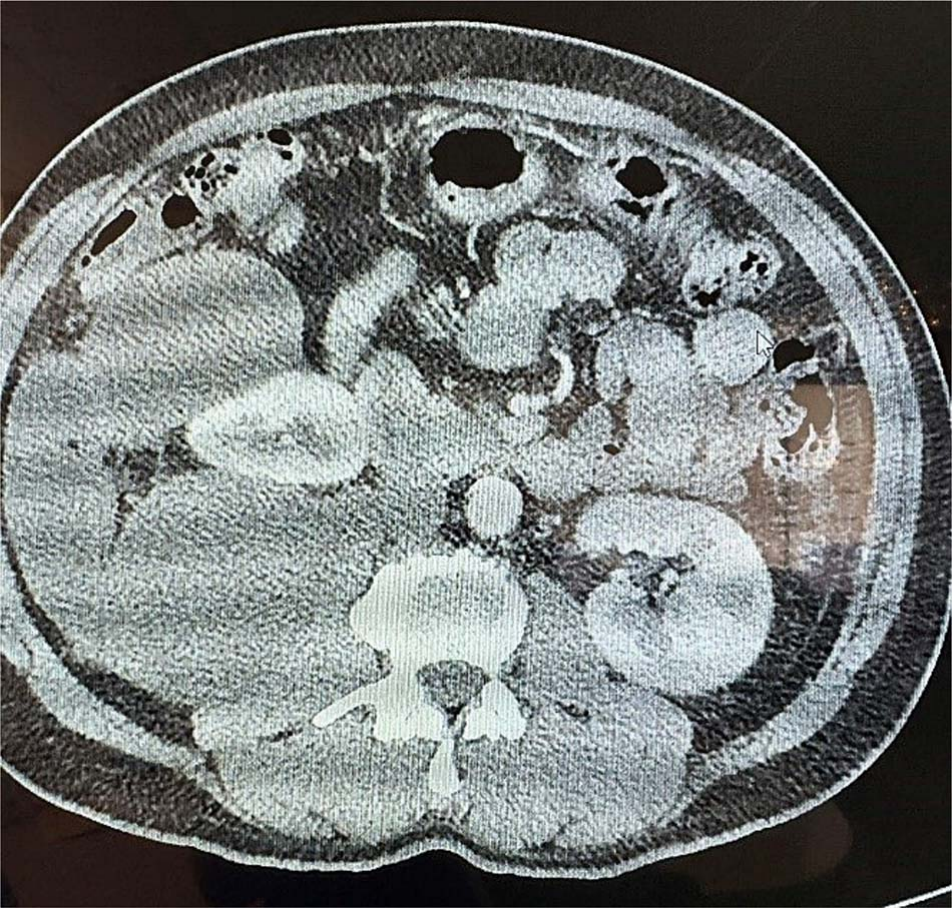
Transverse CT of the mucinous adenocarcinoma.

This is a 53-year-old male with no notable past medical history who presented with a 6-week history of persistent right-sided flank pain. Notably, the patient denied systemic symptoms such as weight loss, nausea, vomiting, or constipation and he denied any urinary symptoms, including dysuria or hematuria. Initial evaluation in the emergency department (ED) included a computed tomography (CT) scan of the abdomen and pelvis, which revealed a 23 cm cystic retroperitoneal mass displacing the right kidney as seen in Figs 1 and 2. The patient was discharged from the ED with a recommendation for follow-up with a surgical specialist.

Upon further evaluation by the surgeon at follow-up, a repeat CT scan was obtained and demonstrated that the mass had grown to 28.5 cm and displayed a lobulated cystic structure with calcifications at its inferior margin. The mass was abutting the cecum and was suspected to be a mucinous neoplasm. Given the concerning size and characteristics of the tumor, surgical resection was recommended.

**Figure 3:**
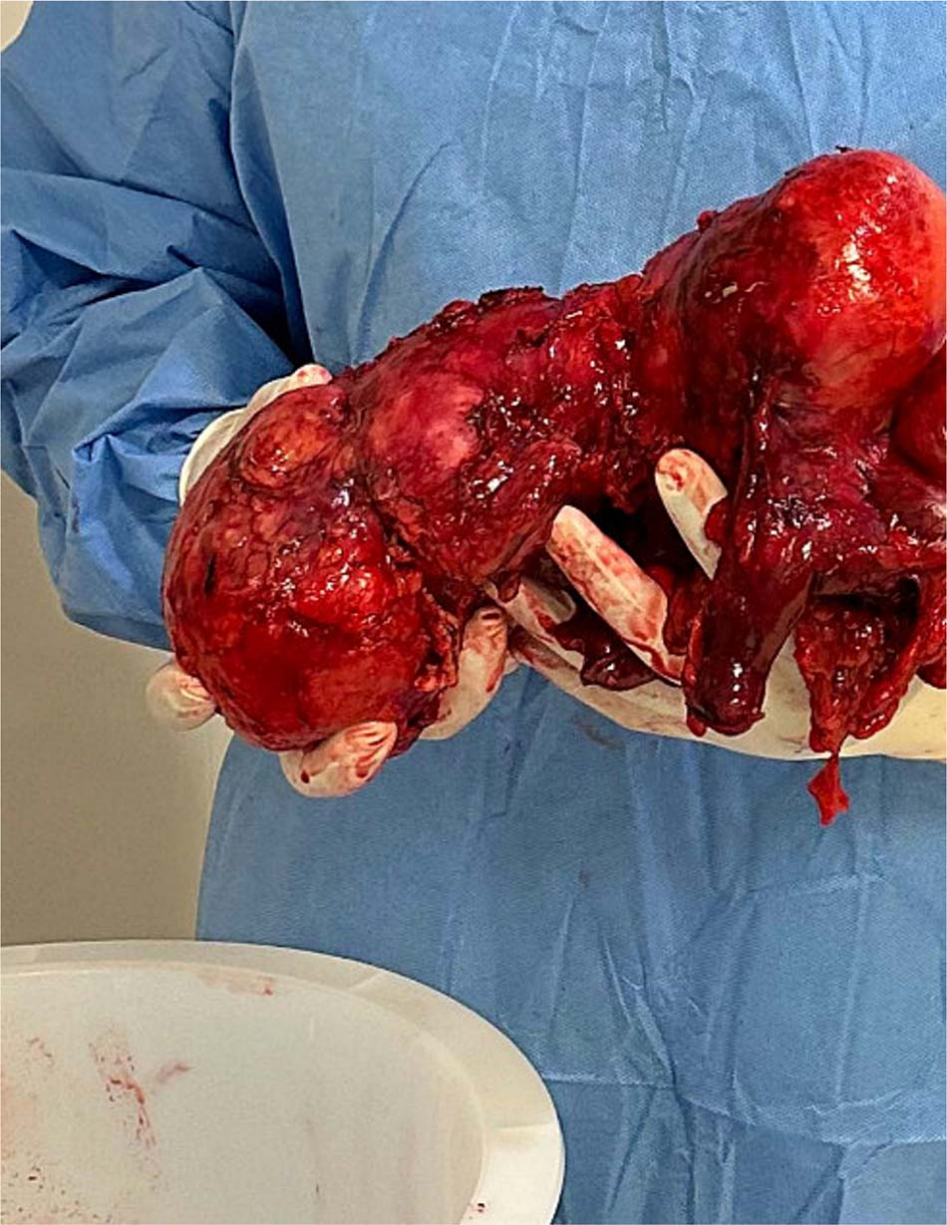
En bloc resected mass.

The surgery was performed via a midline laparotomy. Intraoperatively, the tumor was confirmed to be entirely retroperitoneal, extending from the right pelvic region to the retrohepatic space superiorly. The mass displaced the ascending colon anteriorly and the right kidney anteromedially, but maintained clear planes separating it from the kidney. Given its location and proximity to the cecum, the tumor was resected en bloc along with a right hemicolectomy to ensure complete removal as seen in Fig. 3, and a drain was left in the right paracolic gutter. Postoperatively the drain initially had a large volume output. Drain fluid and urine were sent for analysis to evaluate for urine leakage, but returned negative. The patient was ultimately discharged home on post op day 10, and the drain was eventually removed in clinic.

Initial pathology demonstrated an intact appendiceal mucinous tumor without destructive invasion or involvement of peritoneum, small bowel, or colon. There was no mismatch repair deficiency given normal expression of MLH1, MSH2, MSH6, and PMS2. Nodes were negative for atypia 0/8, with initial grading of T3N0 Stage IIa with negative margins.

The patient was referred to oncology, and 2 months postoperatively underwent a colonoscopy that demonstrated a well appearing ileocolic anastomosis without evidence of inflammation or mucinous tumor recurrence. Three months postoperatively he underwent a surveillance CT scan that showed no residual or metastatic disease, with negative carbohydrate antigen (CA) CA 19–9, CA 125, and carcinoembryonic antigen (CEA) tumor markers. At the 6 month postoperative interval, imaging and labs continued to demonstrate no evidence of metastatic disease. The patient continues to remain disease free at his most current 9 month follow-up.

## Discussion

This case highlights several important aspects of retroperitoneal masses, particularly those that involve large mucinous neoplasms. The differential diagnosis in this case initially included recurrent mucinous neoplasm despite no prior surgical history, which led to the possibility of a primary mucinous adenocarcinoma versus a low-grade appendiceal mucinous neoplasm that could have been present for an extended period [1]. These tumors are typically slow-growing, but their size can cause compression of surrounding organs, as was evident in the urine analysis with concurrent right-sided flank pain [4, 7]. CT imaging, particularly with contrast, plays a pivotal role in assessing the extent of the disease and guiding surgical planning. Calcifications within the tumor, as seen in this case, are often associated with mucinous neoplasms, supporting the suspected diagnosis [4, 8]. Surgical resection remains the definitive treatment for these tumors, especially with rapid growth. In this case, the decision to perform an en-bloc resection with the right hemicolectomy was appropriate, given the tumor's size and its involvement with the cecum. Postoperative management focuses on monitoring for complications, including fluid collections, fistula formations, and infections.

### Treatment

Surgical resection remains the cornerstone of treatment for mucinous adenocarcinoma of the appendix. The type of surgery performed depends on the extent of the disease. For localized tumors, an appendectomy may be sufficient, but for larger/more invasive tumors, procedures that are more extensive are required. With peritoneal spread, cytoreductive surgery combined with hyperthermic intraperitoneal chemotherapy is commonly used [7, 8].

## Conclusion

This case illustrates the complexity of managing giant retroperitoneal mucinous neoplasms. Multidisciplinary care involving surgery, radiology, and pathology is essential for achieving optimal outcomes in patients with large cystic masses that displace critical structures. This case underscores the importance of thorough imaging, careful surgical planning, and vigilant postoperative monitoring, all contributing to a positive recovery. Continued follow-up is crucial, as mucinous tumors, even when resected en bloc, carry a risk of recurrence.
